# Honoring Preferences—The Consistency Between End-of-Life Care and a Veteran’s Goal for Comfort Care

**DOI:** 10.1093/geroni/igaa057.2711

**Published:** 2020-12-16

**Authors:** Joan Carpenter, Winifred Scott, Mary Ersek, Cari Levy, Jennifer Cohen, Mary Beth Foglia, Ciarian Phibbs, Susan Miller

**Affiliations:** 1 Department of Veterans Affairs, Berlin, Maryland, United States; 2 VA Palo Alto Healthcare System, Palo Alto, California, United States; 3 Department of Veterans Affairs, Philadelphia, Pennsylvania, United States; 4 VA Eastern Colorado Health Care System, Aurora, Colorado, United States; 5 VA Puget Sound Health Care System, Seattle, Washington, United States; 6 VA - National Center for Ethics in Health Care, Seattle, Washington, United States; 7 VA Palo Alto Healthcare System, Menlo Park, California, United States; 8 Brown University, Providence, Rhode Island, United States

## Abstract

This study examined the alignment between Veterans’ end-of-life care and a Life-Sustaining Treatment (LST) goal “to be comfortable.” It includes Veterans with VA inpatient or community living center stays overlapping July 2018--January 2019, with a LST template documented by January 31, 2019, and who died by April 30, 2019 (N = 18,163). Using VA and Medicare data, we found 80% of decedents with a comfort care goal received hospice and 57% a palliative care consult (compared to 57% and 46%, respectively, of decedents without a comfort care goal). Using multivariate logistic regression, a comfort care goal was associated with significantly lower odds of EOL hospital or ICU use. In the last 30 days of life, Veterans with a comfort care goal had 43% lower odds (AOR 0.57; 95% CI: 0.51, 0.64) of hospitalization and 46% lower odds of ICU use (AOR 0.54; 95% CI: 0.48, 0.61).

